# Alterations in Supine Position Mobility and Dynamics in Post-Stroke Individuals with Hemiparesis Compared to Neurologically Intact Controls: A Video-Based Observational Assessement

**DOI:** 10.3390/jcm14227949

**Published:** 2025-11-10

**Authors:** Zofia Żukowska, Maciej Krawczyk, Łukasz A. Poniatowski

**Affiliations:** 1 Department of Clinical Physiotherapy, Faculty of Rehabilitation, Józef Piłsudski University of Physical Education, Marymoncka 34, 00-968 Warsaw, Poland; zofia.zukowska9@gmail.com; 2 Department of Neurology, Bielański Hospital, Cegłowska 80, 01-809 Warsaw, Poland; 3 2nd Department of Neurology, Institute of Psychiatry and Neurology, Sobieskiego 9, 02-957 Warsaw, Poland; 4 Department of Neurosurgery, Voivodeship Specialist Hospital in Słupsk, Hubalczyków 1, 76-200 Słupsk, Poland; lukasz.poniatowski@gmail.com

**Keywords:** stroke, supine position, hemiparesis, bed transfer, mobility strategies, rehabilitation

## Abstract

**Background/Objectives:** The aim of this study was a video-based observational assessment of movement strategies during supine position transfers in patients with hemiparesis following a first-ever ischemic stroke. **Methods:** The study included 51 participants (*n* = 51), covering 20 healthy individuals (*n* = 20) and 31 patients (*n* = 31) after their first ischemic stroke with hemiparesis. All participants underwent observational kinematic analysis of supine mobility using video recording and time-lapse analysis. The assessment focused on the time required to complete the task, the number of pelvic movements, the presence of trunk translation, spinal flexion, and pelvic mobility across three planes. **Results:** In the control group, transfers followed a consistent and repetitive sequence in both directions, typically involving trunk translation, spinal flexion, pelvic elevation, and symmetrical movement of both upper and lower limbs. In contrast, post-stroke patients demonstrated altered, asymmetrical, and less efficient movement patterns. These movement strategies were consistent across the hemiparetic group and characterized typical motor responses following stroke. The average transfer time in the study group was approximately three times longer than in the control group. The average number of pelvic movements was 7.2 ± 2.44 in healthy individuals and 16.71 ± 13.52 in post-stroke patients. **Conclusions:** Supine transfers should be routinely assessed in patients after stroke and included as a key focus in physiotherapy goals. The movement patterns required for such transfers represent a distinct component of complex motor function. Both qualitative and quantitative aspects of their execution may have a significant impact on functional independence in individuals with hemiparesis. Identifying typical transfer patterns in hemiparetic patients may offer valuable guidance for early post-stroke rehabilitation planning, particularly in preventing maladaptive compensatory strategies.

## 1. Introduction

Stroke is one of the leading causes of disability and morbidity worldwide [1]. Despite advances in medicine, diagnostics, prevention, and health promotion, increasing life expectancy has resulted in a growing number of stroke survivors living with long-term mobility impairments. This presents a significant challenge for healthcare professionals, who must ensure that individuals with hemiparesis are able to lead active and participatory lives, with independence being the primary goal of rehabilitation [2]. The severity of neurological deficits following stroke, along with the patient’s functional prognosis, is commonly evaluated using the National Institutes of Health Stroke Scale (NIHSS) [3]. Stroke-related musculoskeletal dysfunctions typically include muscle weakness, increased muscle tone, and reduced active and passive joint mobility [4]. One of the most persistent consequences of stroke is upper limb paralysis, affecting up to 80% of patients, particularly hand function, which is essential for performing activities of daily living [5]. Impairments in the upper limb significantly reduce patients’ quality of life and limit their independence [6]. Similarly, lower limb dysfunction can compromise proximal stability, leading to postural asymmetry, difficulties with position changes, impaired upright posture, altered gait patterns, and an elevated risk of falls [7]. In the acute phase of stroke, patients are often bedridden. The term bed mobility or bed independence refers to the ability to roll from a supine to a prone or lateral position and back, sit up on the edge of the bed from a supine position and return, and reposition oneself while lying supine [8]. Clinical observations of stroke patients during hospitalization frequently reveal difficulties in achieving a symmetrical supine position, maintaining a neutral body alignment, repositioning upward, and reaching for nearby objects, such as those on a bedside cabinet [9]. A review of the literature revealed no studies specifically focused on motor strategies or transfer mechanisms in the supine position. This aspect remains undescribed in both healthy individuals and those with hemiparesis, despite being a fundamental component of human motor function. Therefore, based on the authors’ original research protocol, the present study aimed to assess mobility and movement strategies in the supine position among post-stroke patients, while also exploring the potential development of a novel diagnostic assessment tool.

## 2. Materials and Methods

### 2.1. Patient Population

Fifty-one participants (*n* = 51) took part in the study. The control group consisted of 20 employees (*n* = 20) of the Institute of Psychiatry and Neurology in Warsaw who reported no pain or musculoskeletal restrictions and had not undergone spine, abdominal or thoracic surgery in the previous 6 months, covering 14 women (*n* = 14) and 6 men (*n* = 6). The study group comprised 31 patients (*n* = 31), 13 women (*n* = 13) and 18 men (*n* = 18), hospitalized at the 2nd Department of Neurology of the Institute of Psychiatry and Neurology in Warsaw following their first ischemic stroke. The mean age of the control group was 59 years (±8.01 years), while that of the study group was approximately 64.7 years (±13.05 years). Additional demographic details of the study participants are presented in Table 1. Inclusion criteria for the study included the presence of hemiparesis as assessed by the NIHSS, informed consent to participate, absence of musculoskeletal pain, ability to understand commands, and absence of neglect syndrome, superficial or deep sensory deficits, surgical spine stabilization, or abdominal or thoracic surgery in the previous 6 months. Exclusion criteria were lack of informed consent, a subsequent stroke, absence of hemiparesis according to the NIHSS, presence of musculoskeletal pain, inability to understand commands, neglect syndrome, superficial or deep sensory deficits, surgical spine stabilization, abdominal or thoracic surgery in the previous 6 months, or contraindications to the supine position. The study was based on the authors’ supine mobility test protocol. Transfer trials were conducted on a 120 × 200 cm bed and recorded using a video camera with time-lapse imaging. The study was approved by the Senate Committee on Research Ethics of the Józef Piłsudski University of Physical Education (No. SKE 01-53/2022) on 31 March 2022. All participant data has been anonymised. The data is stored on a hard drive at the Institute of Psychiatry and Neurology in Warsaw in accordance with institutional data protection standards for a period of five years.

The authors’ methodology is described as follows:

1. Initial Position: The participant lay supine on the right edge of the bed, with lower limbs extended, feet together, the non-affected limb alongside the body, and the scapula and head resting.
2. Upon the command “START”, participants were permitted to perform any movement toward the left edge of the bed while maintaining a supine position. During the task, participants could flex their lower limbs, move their upper limbs, or lift their head. The task was considered complete when the participant’s body was positioned closest to and parallel with the left edge of the bed in the initial supine position.
3. Subsequently, participants repeated the transfer in the opposite direction, starting from the initial position at the left edge of the bed. The trial was deemed complete when the initial position was regained at the right edge of the bed.
4. Each participant performed the test twice.

In the control group, transfers to the right and left sides were evaluated. For the study group, the sides of the body were defined as “affected” (side with paresis) and “non-affected” (side without established paresis). The analysis of the results included quantitative and qualitative parameters of movement patterns in the supine position (Table 2).

### 2.2. Qualitative Analysis

The camera was mounted on a 2.15 m high tripod, positioned a constant 1 m from the foot end of the bed, allowing it to capture movements of all body segments of the participants. The physiotherapist analyzed the motion on a computer monitor in the standard manner, typical of clinical visual assessment. Basic movements of the lower limbs, upper limbs, neck, and trunk were taken into account. Neck flexion was identified as the movement of the head relative to the stationary upper trunk. Trunk flexion in the sagittal plane was identified as movement of the upper trunk relative to the stationary pelvis. Lateral trunk flexion was identified as movement of the upper trunk relative to the stable pelvis or, vice versa, in the frontal plane. The moment the buttocks left the support surface was clearly visible in the video. Translational movement of the trunk in the frontal plane was identified in the video image as the movement of the shoulder girdle relative to the stationary pelvis and was parallel to the tangent line to the iliac crests. The reliability of the data was assessed using Cohen’s Kappa coefficient and Intraclass Correlation Coefficient (ICC 3.1). The intra-rater reliability of the study was evaluated through a repeat analysis of the results conducted after two years, while the inter-rater reliability was determined based on the assessments of an independent researcher.

### 2.3. Statistical Analysis

Statistical analyses were performed using IBM SPSS Statistics 29.0 (IBM Corp., Armonk, NY, USA) and Microsoft Excel 2010 (Microsoft Corp., Redmond, WA, USA). A significance level of α = 0.05 was adopted. No correction for multiple comparisons was applied, as analyses involved a limited number of predefined movement types within a single experimental framework, consistent with standard practice in neurophysiological research.

## 3. Results

**Figure 1:**
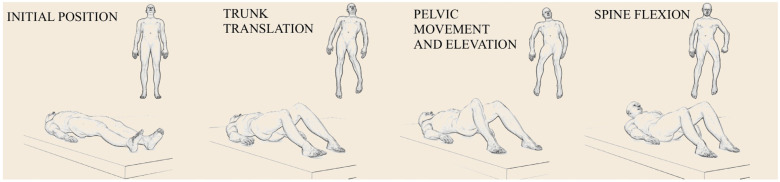
Movement patterns during transfer in supine position performed by healthy individuals. Images in order: initial position, trunk translation, pelvic movement and elevation, spine flexion relative to pelvis.

Based on observations of supine transfers in the control group, consistent movement patterns were identified in both directions. The observed actions included lateral spinal flexion, sagittal spinal flexion, pelvic elevation, and symmetrical limb movements. These strategies were performed fluidly and in a well-coordinated manner. The listed observable components served as the evaluation criteria for transfers in both groups. The approach used by control participants remained consistent across both leftward and rightward directions in terms of qualitative and quantitative parameters. Healthy individuals initiated the task with simultaneous sagittal spinal flexion and lateral trunk translation toward the intended direction. Descriptive statistics indicated that 87.5% of participants exhibited the trunk translation pattern, while 55% displayed spinal flexion. This was followed by a coordinated pelvic elevation, detaching the gluteal region from the bed surface, accompanied by a lateral pelvic shift aligned with the motion of the entire body. Once the pelvis was aligned with the head and thorax, the position of the limbs was adjusted accordingly. This sequence was repeated until the opposite edge of the bed was reached. No use of hand support was observed during the activity. The movement pattern sequence identified during transfers is presented in Figure 1. In contrast, patients who had experienced their first ischemic stroke did not display such consistent movement strategies. A significant difficulty noted was in the planning and coordination of movement during the task. Some participants were unable to complete the transfer. Toward the non-paretic side, the task was unsuccessful in four participants (13%), while toward the paretic side, it failed in five participants (16%). Analysis of the transfer attempts revealed movement strategies typical of individuals with hemiparesis. During transfers toward the paretic side, the following compensatory behaviors were observed: pushing off with the non-paretic upper and lower limbs, rolling toward the affected side, and sagittal spinal flexion while in the lateral lying position. Post-stroke patients initiated repositioning by actively pushing off with their non-paretic limbs. The asymmetrical distribution of muscle strength led to elevation of the pelvis and scapula on the non-affected side, initiating a roll onto the paretic side. In this position, a forceful push and extension of the non-paretic lower limb facilitated pelvic movement toward the target direction, aligning the body parallel to the edge of the bed. This strategy was repeated until the bed edge was reached. During transfers toward the non-affected side, the dominant movement component was pulling with the non-paretic upper limb toward the bed edge. This was accompanied by lateral spinal flexion in the direction of the transfer, with the paretic side of the body following passively. Subsequently, a rotational movement of the pelvis occurred on the non-affected side, along with its elevation from the bed surface. This action enabled patients to realign their body position and initiate the next phase of the transfer. Basic descriptive statistics of the quantitative parameters, presented in Table 3, indicated transfer symmetry in both directions among the control group. The mean time difference between leftward (M = 5.45, SD = 2.31) and rightward (M = 5.30, SD = 1.87) transfers was only 0.15 s. Similarly, the difference in the number of pelvic elevations was 0.2. Based on the data from healthy controls, such deviations were considered normative. In contrast, within the study group, notable differences were observed in both transfer time and the number of pelvic elevations required to complete the task toward the paretic and non-paretic sides. Transfers toward the affected side required, on average, 0.19 more pelvic elevations and took 1.71 s longer compared to transfers toward the non-affected side. Comparison of quantitative parameters (Table 4) revealed statistically significant differences between the control and study groups across all tested variables. Participants in both groups demonstrated improved performance during the second trial, which was subsequently used for qualitative parameter analysis. Descriptive statistics showed that post-stroke patients required approximately three times more time (Me = 24.0, IQR = 24.5) to complete the movement task compared to healthy controls (Me = 9.0, IQR = 6.5). The average number of pelvic elevations during transfers in both directions was—(Me = 6.0, IQR = 3.3) in the control group, whereas patients with hemiparesis required (Me = 12.0, IQR = 10.0) elevations to complete the same task. As indicated in Table 5, a correlation analysis using Spearman’s ρ revealed no statistically significant relationship between the severity of neurological deficits, as assessed by the NIHSS, and the following parameters: total transfer time (*p* = 0.416), time toward the affected side (*p* = 0.183), time toward the non-affected side (*p* = 0.816), total number of pelvic elevations (*p* = 0.463), or the number of elevations toward the affected (*p* = 0.106) and non-affected sides (*p* = 0.934). These findings suggest that the severity of neurological deficits did not significantly influence the quantitative parameters of transfers in the supine position. Spearman’s correlation analyses showed weak, non-significant associations between the severity of neurological deficits (NIHSS) and all movement parameters (ρ = −0.30 to 0.25, *p* = 0.106–0.934). Given the small sample size (*n* = 31), the study was adequately powered only to detect large correlations; the minimum absolute correlation detectable with 80% power (approximated using Pearson correlation formulas) is r ≈ 0.49. Therefore, the absence of significant correlations should be interpreted cautiously. Based on the correlation analysis presented in Table 6, only 11 patients (35%) demonstrated trunk translation toward the affected side, while 20 did not perform this movement. Due to unequal group sizes, the assumption of equal sample sizes was not met, and thus, the Mann–Whitney U test was applied. The results were not statistically significant (U = 76.00, *p* = 0.158, η^2^ = 0.07). The severity of neurological deficits, as measured by the NIHSS, did not affect the likelihood of performing trunk translation toward the affected side. Participants who did not exhibit trunk translation (M = 7.20, SD = 4.23) did not differ significantly in their neurological deficit compared to those who did perform this movement (M = 4.91, SD = 3.11). Similarly, with regard to trunk translation toward the non-affected side, this movement pattern was observed in only 11 individuals (35%) during transfers in that direction (Table 7). The analysis showed no statistically significant difference between the groups (U = 69.00, *p* = 0.088, η^2^ = 0.10). Participants who performed trunk translation toward the non-affected side (M = 4.73, SD = 3.04) did not differ significantly in neurological deficit severity from those who did not employ this strategy (M = 7.30, SD = 4.19). Accordingly, Table 8 and Table 9 present the relationship between the severity of hemiparesis and the presence of sagittal plane spinal flexion during transfers. An independent samples *t*-test was conducted in both cases. For transfers toward the affected side, no statistically significant differences were found between the groups (t (21.64) = −1.54, *p* = 0.134, 95% CI [−5.03, 0.71], d = 0.56). The mean NIHSS score did not differ significantly between patients who did not perform spinal flexion (M = 5.41, SD = 3.30) and those who used this strategy (M = 7.57, S =4.50). In contrast, for transfers toward the non-affected side, the analysis revealed a statistically significant difference between the groups (t (21.64) = −2.09, *p* = 0.049, 95% CI [−5.86, −0.02], d = 0.79). Individuals who performed spinal flexion (M = 8.00, SD = 4.52) exhibited a more severe degree of hemiparesis compared to those who did not engage in this movement during the transfer (M = 5.06, SD = 2.98). According to Cohen’s d, this represents an effect of moderate strength. The severity of hemiparesis, as measured by the NIHSS, did not influence the occurrence of trunk translation in either direction or sagittal spinal flexion toward the affected side. However, higher NIHSS scores were associated with more frequent use of spinal flexion during transfers toward the non-affected side. Subsequent data analysis demonstrated a high degree of consistency. Inter-rater reliability, assessed using Cohen’s Kappa coefficients, ranged from 0.668 to 0.743, indicating moderate to substantial agreement between raters. Intra-rater reliability values were higher (0.801–0.870), corresponding to substantial to almost perfect agreement (Table 10). The intraclass correlation coefficient (ICC) for the number of pelvic elevations indicated a high level of agreement (>0.9), reflecting excellent reliability (Table 11).

## 4. Discussion

The presented study addresses the issue of mobility in the supine position within the bed among individuals with hemiparesis. Due to the lack of detailed descriptions of movement patterns in healthy individuals, a control group was included. Based on the observations of transfers, normative ranges were established, and the movement strategies employed by healthy participants were identified. Individuals without pain or musculoskeletal limitations demonstrated selectively generated, multiplanar, and coupled movements. These involved all spinal segments across various planes, as well as coordinated movements of the upper and lower limbs, exhibiting relatively high repeatability. Selective internal movements of the trunk refer to the coordinated action of trunk muscles to perform specific movements like flexion, extension, rotation, and lateral bending, which are fundamental for posture and daily activities [10]. These movements rely on the interplay of various muscles. A healthy motor system relies on the ability to dissociate or independently control the trunk and pelvis for precise movement, and a breakdown in this dissociation indicates a loss of selective motor control [11]. Focal brain injury not only weakens trunk muscles but also impairs the coordinated control required for symmetrical movement [12]. The movements we identified in healthy individuals in our study, while lying on their backs, were asymmetrical and three-dimensional, following repetitive patterns. Patients with hemiparesis performed these movements in a completely different way, and their movement patterns were impoverished. The order of muscle activity in the human trunk depends on mechanisms of the central nervous system that we do not yet understand. Trunk muscle synergies in healthy individuals appear to be consistent and comparable across subjects [13]. Trunk movements occur within small ranges due to anatomical limitations. Each movement is the sum of smaller motions taking place in individual intervertebral joints. This requires subtle and precise coordination of muscle contractions on the left and right sides at the same time. Importantly, the contraction of agonist muscles is accompanied by the contraction of antagonist muscles with varying amplitudes. This ensures proper coactivation [10]. Therefore, weakness of the muscles on one side of the trunk results in very poor mobility performance in the horizontal position, which becomes most apparent during asymmetrical activities. One such activity is moving while lying on the back. Moreover, it should be noted that every voluntary movement of the trunk requires stabilization, which, in the case of hemiparesis, is reduced or even impossible. For example, during lateral translation of the upper trunk, a stable pelvis is necessary, which is made possible by the muscles surrounding the hip joint.

The analysed quantitative parameters, including the time required and the number of pelvic elevations during transfers to the left or right, also proved to be reproducible across trials in our study. Notable and fundamental differences in movement patterns were observed between healthy individuals and those with hemiparesis. The selective control of trunk and pelvic motion during transfers, as observed in the control group, was complex and biomechanically demanding. In patients with hemiparesis, this control was impaired, necessitating compensatory motor strategies based on the individual’s functional abilities. In some cases, completion of the task proved impossible. Observations of post-stroke patients, evaluated using the authors’ original protocol, revealed movement strategies employed with varying frequency and scope during supine mobility. These findings were supported by high inter- and intra-rater reliability values, confirming the consistency and replicability of the observed movement patterns. Strategies used when transferring toward the side unaffected by paresis included active pulling with the contralateral upper limb, lateral spinal flexion toward the target direction, and pelvic rotation in the horizontal plane. Pelvic elevation and horizontal movement on the non-affected side were significantly more effective than on the paretic side, likely due to weakness in the hip extensors, abdominal muscles, and spinal muscles on the affected side. The extent to which these strategies were employed appeared to depend on the severity of the neurological deficit and the functionality of the affected limbs. In contrast, transfers toward the paretic side were characterized by pushing off with the unaffected lower and upper limbs, rolling onto the affected side, and sagittal spinal flexion while lying laterally.

To objectively assess the severity of hemiparesis among study participants, the NIHSS was applied. Based on this scale, neurological status is categorized as mild (1–5 points), mild to moderate (5–14 points), severe (15–24 points), or very severe (>25 points) [14]. The NIHSS is a validated, reliable, and widely used tool for evaluating the severity of neurological deficits [15]. It also serves as a predictor of long-term functional outcomes and patient independence [12]. In the present study, no significant relationship was observed between the severity of neurological impairment and the quantitative or qualitative parameters of supine transfers. However, based on these findings alone, it cannot be concluded that the severity of neurological deficits has no impact on mobility skills or movement selectivity. Further research involving a larger and more diverse cohort of individuals with hemiparesis is warranted. Specifically, including post-stroke patients with a broader range of NIHSS scores may allow for a more accurate determination of the relationship between neurological impairment and the movement strategies employed during supine transfers. The ability to perform transfers plays a crucial role in daily functioning, enabling safe positional changes and facilitating increased activity levels. Effective transfer skills ultimately improve patient independence. In recent years, an increasing number of studies have focused on transfers in post-stroke individuals, highlighting their importance in neurological rehabilitation [16,17,18]. For example, Osada et al. analyzed motor task performance during gait initiation immediately following rising from a seated position (sit-to-walk) [19]. Their findings revealed that difficulty in initiating smooth gait after standing was strongly correlated with impaired balance in this population. The authors concluded that successful task execution requires gait initiation before full trunk extension. Another frequently studied transfer is the sit-to-stand transition, as examined by Silva et al. [20]. Their results indicated poorer performance in the post-stroke group compared to healthy individuals. Moreover, kinematic changes in trunk movement during this task were linked to decreased efficiency in generating and transferring torque by the trunk flexor muscles.

Participants in the study group employed multiple compensatory strategies during this activity, including asymmetrical weight distribution, leaning toward the unaffected side, and uneven torque distribution across the knee joints. Post-stroke patients required significantly more time to complete the STS (sit-to-stand) task compared to the control group, a finding the authors attributed to sensorimotor impairments in this population. Moreover, prolonged STS completion time was associated with a higher risk of falls. In a study by Sunnerhagen et al., two functional motor tasks, transitioning from a supine to a seated position and transferring from a seated position to a chair, were presented as reliable clinical tests of mobility in patients with hemiparesis [21]. Several studies have described transfers from the supine position, particularly rolling from a supine to a lateral position. In research conducted by Kafri et al., the focus was on the activation and efficiency of specific trunk muscles during this task in individuals with hemiparesis [22]. Their analysis showed that rolling toward the non-affected side posed greater difficulty for post-stroke patients than rolling toward the paretic side. Meanwhile, studies by Chiang et al. explored the correlation between kinematic variables and trunk control quality during lateral rolling from a supine position [23]. They identified disrupted rolling patterns compared to healthy individuals, emphasizing the potential of lateral rolling assessments for evaluating post-stroke patients and guiding physiotherapeutic strategies. A range of scientific studies has focused on transfers in post-stroke patients. Analysis of gait, clinical tests, and positional change abilities enables the identification and evaluation of challenges faced by this population. However, no previous study has described supine-position transfers in post-stroke patients, nor have normative standards for healthy individuals been established. A more detailed description and deeper understanding of these mechanisms could contribute to the development of more effective rehabilitation strategies and enhance patient mobility in both low and high positions. This research provides new insights into the functional status of individuals after stroke. Defining and identifying limitations in mobility and movement strategies in the supine position presents a novel challenge in the field of physiotherapy. The transfer task may serve as a diagnostic tool, a rehabilitation goal, or a component of therapy. The impact of supine transfers on other motor activities restored to pre-disease levels, as well as on the movement selectivity required for mobility in the supine position and overall functional activity, remains unclear. Mobility in the supine position among individuals with hemiparesis should be re-educated, encompassing both the complete task and the targeted activation of the muscle groups involved. Special attention should be given to the role of proximal axial muscles, including those of the abdomen, back, neck, and lower limbs, which contribute to the execution of these movement sequences. At present, it is not possible to definitively identify which muscle groups are most critical for the task as a whole. It is plausible that in individuals with a “strong trunk”, transfers toward both the affected and non-affected sides could be more efficient, despite severe paresis of the upper or lower limbs. This hypothesis, among others, warrants further investigation in future research.

## 5. Conclusions

It should be considered to place greater emphasis on the re-education of selective trunk muscle activity on the paretic side in stroke patients. For individuals with hemiparesis, it is recommended that supine mobility be re-educated using a dual approach: addressing the task as a whole and selectively targeting the muscle groups involved.

## Figures and Tables

**Table 1:** Demographic and clinical characteristics of patients.

	Study Group (*n* = 31)	Control Group (*n* = 20)
Mean age [years]	64.71	58.75
Median age [years]	67	59
Standard deviation of age	13.05	8.01
Number of women [*n*]	13	14
Number of men [*n*]	18	6
Right hemiplegia [*n*]	19	/
Left hemiplegia [*n*]	12	/
Mean NIHSS [0–42]	6.39	/
Median NIHSS [0–42]	5	/
Standard deviation of NIHSS	3.97	/

**Table 2:** Quantitative and qualitative parameters assessed during transfers.

Quantitative Parameter	Time	Total transfer time and its components; measured in seconds
	Number of Pelvic Elevations	Total number of pelvic elevations and their components
Qualitative Parameter	Trunk Translation	Lateral spinal flexion relative to a stabilized pelvis in the frontal plane
	Spinal Flexion Relative to Pelvis	Lift of the head, thoracic segment, and scapulae from the bed in the sagittal plane
	Pelvic Movement	Pelvic activity in the sagittal, frontal, and horizontal planes; assessed separately in each plane

**Table 3:** Basic descriptive statistics of the studied variables with Shapiro–Wilk test.

Variables	M	Me	SD	Sk.	Kurt.	Min.	Max.	W	*p*
TL	5.45	5.00	2.31	0.33	−1.11	2,00	9.00	0.90	0.041
TP	5.30	5.00	1.87	0.48	−1.31	3.00	8.00	0.85	0.004
PEL	3.70	3.00	1.34	0.32	−1.19	2.00	6.00	0.88	0.020
PER	3.50	3.00	1.28	1.43	2.17	2.00	7.00	0.82	0.002
TA	18.55	15.00	19.39	2.76	8.38	5.00	93.00	0.67	<0.001
TNA	16.84	12.00	14.18	1.81	2.88	4.00	55.00	0.77	<0.001
PEA	8.45	7.00	7.46	3.78	17.84	3.00	44.00	0.62	<0.001
PENA	8.26	7.00	6.53	2.41	7.59	3.00	34.00	0.76	<0.001

TL—time in left side, TP—time in right side, PEL—number of pelvis elevations in left side, PER—number of pelvis elevation in right side, TA—time in affected side, TNA—time in non-affected side, PEA—number of pelvis elevations in affected side, PENA—number of pelvic elevations in non-affected side.

**Table 4:** Comparison of quantitative parameters (total transfer time, total number of pelvic elevations) between the study and control groups. PE—number of pelvic elevations during transfer.

	Study Group (*n* = 31)			Control Group (*n* = 20)								
Dependent Variable	Mean Rank	Median Me	IQR	Mean Rank	Median Me	IQR	Z	*p*	η^2^	HL Median Diff	95% CI Low	95% CI High
Total time trial I	34.50	28.0	25.5	12.83	9.5	7.0	5.08	<0.001	0.52	17	9	28
Total time trial II	33.77	24.0	24.5	13.95	9.0	6.5	4.65	<0.001	0.43	15	8	26
PE trial I	33.77	15.0	13.5	13.95	7.0	3.5	4.65	<0.001	0.43	8	4	12
PE trial II	33.32	12.0	10.0	14.65	6.0	3.3	4.38	<0.001	0.38	6	4	11

**Table 5:** Correlation of paresis severity, expressed by NIHSS, with total time, total number of pelvic elevations, time toward the non-affected side, time toward the affected side, number of pelvic movements toward the non-affected side, and number of pelvic movements toward the affected side in the study group.

Variable		NIHSS
Total transfer time	Spearman’s ρ	0.15
	significance	0.416
Total number of pelvic elevations	Spearman’s ρ	−0.14
	significance	0.463
Time in affected side	Spearman’s ρ	0.25
	Significance	0.183
Time in non-affected side	Spearman’s ρ	0.04
	Significance	0.816
Number of pelvic elevations in non-affected side	Spearman’s ρ	−0.30
	Significance	0.106
Number of pelvic elevations in affected side	Spearman’s ρ	0.02
	Significance	0.934

**Table 6:** Correlation of paresis severity, expressed by NIHSS with the occurrence of trunk translation toward the affected side.

	Individuals Who Did Not Perform Trunk Translation (*n* = 20)				Individuals Who Performed Trunk Translation (*n* = 11)						
Dependent Variable	Mean Rank	M	Me	SD	Mean Rank	M	Me	SD	Z	*p*	η^2^
NIHSS	17.70	7.20	6.50	4.23	12.91	4.91	5.00	3.11	1.40	0.164	0.07

**Table 7:** Correlation of paresis severity, expressed by NIHSS with the occurrence of trunk translation toward the non-affected side.

	Individuals Who Did Not Perform Trunk Translation (*n* = 20)				Individuals Who Performed Trunk Translation (*n* = 11)						
Dependent Variable	Mean Rank	M	Me	SD	Mean Rank	M	Me	SD	Z	*p*	η^2^
NIHSS	18.05	7.30	7.00	4.19	12.27	4.73	5.00	3.04	−1.70	0.088	0.10

**Table 8:** Correlation of paresis severity, expressed by NIHSS with the occurrence of spinal flexion toward the affected side.

	Individuals Who Did Not Perform Spinal Flexion (*n* = 17)				Individuals Who Performed Spinal Flexion (*n* = 14)						
Dependent Variable	Mean Rank	M	Me	SD	Mean Rank	M	Me	SD	Z	*p*	η^2^
NIHSS	14.3	5.41	5.00	3.30	18.0	7.57	5.5	4.50	−1.13	0.263	0.043

**Table 9:** Correlation of paresis severity, expressed by NIHSS with the occurrence of spinal flexion toward the non-affected side.

	Individuals Who Did Not Perform Spinal Flexion (*n* = 17)				Individuals Who Performed Spinal Flexion (*n* = 14)						
Dependent Variable	Mean Rank	M	Me	SD	Mean Rank	M	Me	SD	Z	*p*	η^2^
NIHSS	13.6	5.06	5	2.97	18.9	8.00	6.5	4.52	−1.63	0.106	0.088

**Table 10:** Values of Cohen’s Kappa coefficient for inter- and intra-rater reliability in the assessment of spinal flexion and trunk translation movements.

Movement Type	Inter-Rater	Intra-Rater
Spinal flexion in affected side	0.743	0.808
Spinal flexion in non-affected side	0.676	0.870
Trunk translation in affected side	0.729	0.801
Trunk translation in non-affected side	0.668	0.859

**Table 11:** Inter- and intra-rater reliability assessed using Intraclass Correlation Coefficient (ICC 3.1) for number of pelvis elevations in affected side (PEA) and number of pelvic elevations in non-affected side (PENA).

	Inter-Rater				Intra-Rater			
**Movement Type**	**ICC**	**F**	**Pval**	**Cl 95%**	**ICC**	**F**	**Pval**	**Cl 95%**
PEA	0.9997	6755.4	<0.0001	[1.0, 1.0]	0.9884	172.1	<0.0001	[0.98, 0.99]
PENA	0.9996	5397.8	<0.0001	[1.0, 1.0]	0.9932	292.7	<0.0001	[0.99, 1.0]

## Data Availability

The datasets used and/or analysed during the current study are available from the corresponding author on reasonable request.
